# Complete Genome Sequences of 15 Chikungunya Virus Isolates from Puerto Rico

**DOI:** 10.1128/genomeA.00561-17

**Published:** 2017-07-06

**Authors:** Wes A. Sanders, Kristin Long, Brenda Rivera, Heather A. Vincent, Mark T. Heise, Jose F. Rodriguez-Orengo, Nathaniel J. Moorman

**Affiliations:** a Department of Microbiology and Immunology, University of North Carolina, Chapel Hill, North Carolina, USA; b UNC Lineberger Comprehensive Cancer Center, Chapel Hill, North Carolina, USA; c Department of Genetics, University of North Carolina, Chapel Hill, North Carolina, USA; d Department of Health, Epidemiology Division, University of Puerto Rico, San Juan, Puerto Rico; e Department of Biochemistry, School of Medicine, University of Puerto Rico, San Juan, Puerto Rico; f Fundacion de Investigacion de Diego, San Juan, Puerto Rico

## Abstract

Here, we report the complete genome sequences of 15 chikungunya virus strains isolated from human plasma from infected patients in Puerto Rico. The results show that currently circulating chikungunya strains in Puerto Rico are closely related.

## GENOME ANNOUNCEMENT

Chikungunya virus (CHIKV) is an arthropod-borne alphavirus that causes febrile illness associated with severe acute and persistent arthralgia (1). Recent CHIKV outbreaks in the Indian Ocean and the introduction of the virus into the Americas in 2013 illustrate CHIKV’s reemergence as a global threat to public health (2). Currently, there are no licensed vaccines or virus-specific therapies for treating acute or chronic CHIKV disease (3). Thus, there is an urgent need for a better understanding of the factors that contribute to CHIKV pathogenesis in order to develop safe and effective vaccines and therapies.

CHIKV sequence diversity in currently circulating strains of CHIKV could impact the efficacy of novel therapeutics targeting viral proteins. To determine the genetic diversity of CHIKV in the current epidemic in the Americas, we sequenced multiple CHIKV strains isolated from infected patients in Puerto Rico. Human serum samples from patients positive for CHIKV by quantitative reverse transcriptase PCR were obtained by the University of Puerto Rico. CHIKV infectious virus was amplified from serum samples by a single passage on C6/36 mosquito cells. The virus was purified from cell supernatants by centrifugation through a sucrose cushion. Genomic RNA was extracted from sucrose-purified virions using TRIzol (Thermo Fisher Scientific). Virion RNA was reverse-transcribed using random primers (Superscript II; Invitrogen) and converted to double-stranded DNA using the NEBNext mRNA second-strand synthesis module (New England Biolabs). Sequencing libraries were prepared using the Nextera XT DNA library preparation kit (Illumina) and sequenced using a MiSeq version 3 reagent kit (150 cycle) (Illumina). Total reads were mapped to the CHIKV genome using Bowtie2 version 2.3.1 and a GenBank reference genome (accession no. EF452493.1). We obtained an average coverage depth of >10 at each nucleotide. The consensus genome sequences were derived from the most common nucleotide present at each coordinate.

### Accession number(s).

The complete genomic sequences for the 15 chikungunya virus genomes reported here have been deposited in GenBank under the accession numbers MF001505, MF001506, MF001507, MF001508, MF001509, MF001510, MF001511, MF001512, MF001513, MF001514, MF001515, MF001516, MF001517, MF001518, and MF001519.
